# P-538. Epidemiology and Clinical Characteristics of Hand Foot and Mouth Disease of Children and Adolescence Aged < 18 Years Old During Outbreak in Jakarta, Indonesia

**DOI:** 10.1093/ofid/ofaf695.753

**Published:** 2026-01-11

**Authors:** Nina Dwi Putri, Pratama Wicaksana, Aqila Sakina Zhafira, Ageng Wiyatno, Nanda Ayu Syavira, Rini Fajarani, Indri Nethalia

**Affiliations:** Faculty of Medicine, Universitas Indonesia, Jakarta, Jakarta Raya, Indonesia; Universitas Indonesia, Jakarta, Jakarta Raya, Indonesia; Pediatrics Tropical Disease and Infection Research and Innovation, Jakarta, Jakarta Raya, Indonesia; National Research and Innovation Agency (BRIN), Bogor, Jawa Barat, Indonesia; Pediatrics Tropical Disease and Infection Research and Innovation, Jakarta, Jakarta Raya, Indonesia; Universitas Indonesia, Jakarta, Jakarta Raya, Indonesia; Pediatrics Tropical Disease and Infection Research and Innovation, Jakarta, Jakarta Raya, Indonesia

## Abstract

**Background:**

HFMD has become a public health issue in Southeast Asia, with Indonesia experiencing outbreaks post-pandemic. While various enteroviruses cause HFMD, EV71 is linked to severe cases in neighbouring countries. A recent seroprevalence study for EV71 revealed a 99.6% positivity rate, but limited data exists on EV71 severe cases. In the acute flaccid paralysis surveillance, non-polio enterovirus also contributed to the aetiology. Therefore, this study aims to understand the epidemiology of HFMD cases in Indonesia to support data for EV71 vaccine implementation.

**Methods:**

This observational study aimed to identify pathogens in clinically diagnosed HFMD cases. Participants (aged 6 months to < 18 years) exhibiting HFMD signs and symptoms were recruited from 17 hospital-based and community-based research sites in Jakarta. Stool and/or vesicle or nasopharyngeal or buccal or cerebrospinal fluid (CSF) were collected from participants. Enterovirus family detection was performed on these samples using polymerase chain reaction (PCR), followed by phylogenetic analysis via Sanger sequencing for further characterization.

**Results:**

Participant recruitment was conducted between March and August 2024, 83.5% was children 6 months to 5 years old. Analysis of 176 samples revealed that 85 (48.3%) were positive for enterovirus families. Subsequent sequencing identified Coxsackievirus A6 (CVA6) as the predominant serotype (39.2%), followed by CVA16 (1.7%), CVA19 (1.1%), and CVA24, Poliovirus 2, Rhinovirus A, EV84, and Human Rhinovirus C, each detected in 0.6% of positive samples. Notably, 3.4% of enterovirus-positive samples yielded unidentifiable viral sequences. The most frequent clinical manifestations in participants with positive PCR results included mucocutaneous lesions on the hands, feet, or mouth (46%), fever (43.7%), oropharyngeal pain or sore throat (23.9%), cough (15.3%), nausea (7.9%), seizure (7%), fatigue/malaise (6.8%), diarrhea (3.4%), and abdominal pain (1.7%). Seizure was linked to CVA6 (five subjects) and CVA16 (1 subject) infections.

**Conclusion:**

In this study, CVA6 was the predominant aetiology and linked to more severe cases, while EV71 was not detected in this cohort. Further comprehensive surveillance is warranted to detect severe and lethal cases.

**Disclosures:**

Nina Dwi Putri, MD, MSc, Sinovac: Grant/Research Support

